# Temporal Variation of the Skin Bacterial Community and *Batrachochytrium dendrobatidis* Infection in the Terrestrial Cryptic Frog *Philoria loveridgei*

**DOI:** 10.3389/fmicb.2017.02535

**Published:** 2017-12-22

**Authors:** Mariel Familiar López, Eria A. Rebollar, Reid N. Harris, Vance T. Vredenburg, Jean-Marc Hero

**Affiliations:** ^1^Environmental Futures Research Institute, School of Environment, Griffith University, Gold Coast, QLD, Australia; ^2^Departamento de Ecología Evolutiva, Instituto de Ecología, Universidad Nacional Autónoma de México, Mexico City, Mexico; ^3^Department of Biology, James Madison University, Harrisonburg, VA, United States; ^4^Amphibian Survival Alliance, London, United Kingdom; ^5^Department of Biology, San Francisco State University, San Francisco, CA, United States; ^6^School of Science and Engineering, University of the Sunshine Coast, Maroochydore, QLD, Australia

**Keywords:** chytridiomycosis, skin bacteria, amphibians, *Philoria loveridge*, bacteria diversity

## Abstract

In animals and plants, symbiotic bacteria can play an important role in disease resistance of host and are the focus of much current research. Globally, amphibian population declines and extinctions have occurred due to chytridiomycosis, a skin disease caused by the pathogen *Batrachochytrium dendrobatidis* (Bd). Currently amphibian skin bacteria are increasingly recognized as important symbiont communities with a relevant role in the defense against pathogens, as some bacteria can inhibit the growth of *B. dendrobatidis*. This study aims to document the *B. dendrobatidis* infection status of wild populations of a terrestrial cryptic frog (*Philoria loveridgei*), and to determine whether infection status is correlated with changes in the skin microbial communities. Skin samples of *P. loveridgei* were collected along an altitudinal range within the species distribution in subtropical rainforests in southeast Australia. Sampling was conducted in two years during two breeding seasons with the first classified as a “La Niña” year. We used Taqman real-time PCR to determine *B. dendrobatidis* infection status and 16S amplicon sequencing techniques to describe the skin community structure. We found *B. dendrobatidis*-positive frogs only in the second sampling year with low infection intensities, and no correlation between *B. dendrobatidis* infection status and altitude, frog sex or size. Skin bacterial diversity was significantly higher in *P. loveridgei* frogs sampled in the 1st year than in the 2nd year. In addition, 7.4% of the total OTUs were significantly more abundant in the 1st year compared to the 2nd year. We identified 67 bacterial OTUs with a significant positive correlation between infection intensity and an OTU’s relative abundance. Forty-five percent of these OTUs belonged to the family Enterobacteriaceae. Overall, temporal variation was strongly associated with changes in *B. dendrobatidis* infection status and bacterial community structure of wild populations of *P. loveridgei*.

## Introduction

Symbiotic relationships between bacteria and macro-organisms are ubiquitous in nature. The study of symbiotic interactions is a well-established field, yet recently has received increased attention due to the development of next generation sequencing technologies. Specifically, the role of beneficial bacterial communities has been the focus of research for wildlife and human hosts, as they can play important roles providing protection against pathogens (Harris et al., 2009; Jiménez and Sommer, 2016; Walke and Belden, 2016). In amphibians, some bacterial species on their skin inhibit the growth of pathogenic fungi (Harris et al., 2006; Becker et al., 2015; Woodhams et al., 2015) and have shown to play a protective role against chytridiomycosis, a deadly skin fungal disease (Harris et al., 2009; Becker and Harris, 2010; Holden et al., 2015).

Worldwide, many amphibian species are facing significant declines and extinctions attributable to chytridiomycosis (Collins and Storfer, 2003; Stuart et al., 2004; Wake and Vredenburg, 2008; Wake, 2012), the infectious diseases caused by *Batrachochytrium dendrobatidis* (Bd) and *B. salamandrivorans* (Bsal) (Berger et al., 1998; Longcore et al., 1999; Skerratt et al., 2007; Wake and Vredenburg, 2008; Martel et al., 2013). Interestingly, amphibian species exhibit differential susceptibility to Bd infections, which is reflected in the worldwide variation in mortality due to Bd and in Bd intensity and prevalence among species from different habitats, life histories and taxonomic affinities (reviewed in Skerratt et al., 2007; Kilpatrick et al., 2010). These variations are not completely understood, with research suggesting species-specific physiological and ecological traits, such as presence of symbiotic bacterial communities, differential immune response, production of antimicrobial peptides, behavioral patterns and environmental conditions (Kriger and Hero, 2008; Jiménez and Sommer, 2016; Rebollar et al., 2016a).

Given the beneficial role in disease resistance of symbiotic bacterial communities, recent attention has been given to the interaction occurring between amphibian skin microbiota and Bd. Recent evidence indicates that diversity and structure of skin bacterial communities is influenced by several factors such as species-specific traits (McKenzie et al., 2012; Rebollar et al., 2016b; Assis et al., 2017), developmental changes (Kueneman et al., 2014, 2016; Longo et al., 2015; Longo and Zamudio, 2017b), geographic location (Belden et al., 2015; Longo and Zamudio, 2017b; Muletz Wolz et al., 2017). Moreover, skin bacterial diversity is influenced by the microbial communities present in the environment (Fitzpatrick and Allison, 2014; Loudon et al., 2014, 2016; Walke et al., 2014; Rebollar et al., 2016b) as well as by pathogen infection (Jani and Briggs, 2014; Walke et al., 2015; Longo and Zamudio, 2017a).

It remains unclear how climatic conditions influence the composition of microbial communities on the amphibian skin (Kueneman et al., 2014; Jiménez and Sommer, 2016; Longo and Zamudio, 2017a; Medina et al., 2017; Muletz Wolz et al., 2017; Sabino-Pinto et al., 2017). However, in some amphibian species correlation between the variation in amphibian microbiota and abiotic factors (air temperature, water temperature, pH, conductivity, seasonality) has been reported (Kueneman et al., 2014; Krynak et al., 2015, 2016; Longo et al., 2015; Longo and Zamudio, 2017a; Muletz Wolz et al., 2017). For example, in *Lithobates yavapaiensis*, an increase in alpha diversity (Shannon index) of skin bacteria from summer to winter was found, as was a difference in community composition between seasons (Bray–Curtis dissimilarities) (Longo et al., 2015). Similarly, the bacterial composition of *Eleutherodactylus coqui* changed between seasons, potentially allowing frogs to limit Bd infection in the warm wet season (Longo and Zamudio, 2017a).

In addition to the potential role that climatic conditions and abiotic factors have on skin microbiota, Bd prevalence and infection has also been found to be influenced by these factors. Several environmental factors such as altitude, temperature and moisture have been associated with chytridiomycosis outbreaks (Berger et al., 2004; Drew et al., 2006; Lips et al., 2006; Kriger and Hero, 2007, 2008; Kriger et al., 2007; Longo et al., 2010). For example, several studies have shown a decrease in severity of infection at warmer temperatures and or dryer seasons (Retallick et al., 2004; Woodhams and Alford, 2005; Kriger and Hero, 2007; Kriger et al., 2007; Rowley and Alford, 2013). Numerous studies suggest that chytridiomycosis presence, prevalence or intensity can be higher under cooler conditions (Berger et al., 2004; McDonald et al., 2005; Ouellet et al., 2005; Drew et al., 2006; Kriger and Hero, 2007; Kriger et al., 2007; Longo et al., 2010) and/or high elevation (Bosch et al., 2001; Burrowes et al., 2004; McDonald et al., 2005; Woodhams and Alford, 2005; Lips et al., 2006; Frías-Alvarez et al., 2008; Walker et al., 2010). Collectively, these studies support the hypothesis that amphibian populations declines associated with chytridiomycosis occur predominantly at high elevations and during cooler conditions. This pattern, along with the preference of Bd for relatively cool and humid conditions for growth in culture of approximately 23°C, suggests that environmental temperature and humidity are likely to be an important factor in disease dynamics (Muths et al., 2008).

Moreover, studies on Australian amphibian species have revealed that Bd infection is strongly associated with aquatic habitats (Kriger and Hero, 2007) and in frog species that hibernate in aquatic microhabitats rather than terrestrial (Skerratt et al., 2010). This pattern of aquatic amphibian species presenting higher infection and prevalence rates has also been reported among other amphibian species worldwide and has led researchers to hypothesize that terrestrial species may avoid Bd infection through microhabitat protection (Berger et al., 1998; Lips et al., 2006; Kriger and Hero, 2007; Brem and Lips, 2008; Catenazzi et al., 2011). Whilst Bd is more associated with aquatic habitats, terrestrial amphibians can also be infected with Bd, with some species experiencing population declines (Berger et al., 1998; Bell et al., 2004; Burrowes et al., 2004; Lips et al., 2006; Kriger and Hero, 2007; Brem and Lips, 2008; Longo and Burrowes, 2010; Catenazzi et al., 2011). To the best of our knowledge, only a few Australian terrestrial species have been reported with Bd infections. Negative Bd infection was reported on terrestrial Australian microhylids of the Wet Tropics (Hauselberger and Alford, 2012). However, Bd infection was reported for *Cophixalus ornatus* and *Assa darlingtoni* (Kriger and Hero, 2006, 2007). These limited and inconclusive results warrant further investigation of Bd suceptibility in Australian terrestrial amphibians, and the role of skin microbiota on host-pathogen interactions.

*Philoria loveridgei* is a microendemic, diurnal terrestrial frog with terrestrial and nidicolous development to metamorphosis (embryos develop in the broken-down jelly capsules in a nest basin in mud, but they do not go to water) (Knowles et al., 2004; Anstis, 2013). This species is among the rarest vertebrates in eastern Australia, listed as vulnerable due to their habitat specialization and restricted mountaintop distribution (Hines et al., 1999; Knowles et al., 2004). Frogs from this fossorial species are small and highly cryptic making them difficult to find. Males construct shallow burrows under moist forest leaf litter or rocks, where they emit a soft mating call during breeding season to attract females. Inside this nest inguinal amplexus, egg laying and larval metamorphosis occur (Hines et al., 1999; Knowles et al., 2004; Anstis, 2013).

A few studies have addressed the role of climatic changes and temporal variation on host–pathogen interactions of amphibians including the role of skin microbiota and Bd (Jani and Briggs, 2014; Longo et al., 2015; Longo and Zamudio, 2017a; Medina et al., 2017; Muletz Wolz et al., 2017; Sabino-Pinto et al., 2017). However, temporal variation in these studies has been related to changes in seasons within the same year (Longo et al., 2015; Longo and Zamudio, 2017a; Muletz Wolz et al., 2017; Sabino-Pinto et al., 2017). Therefore, to better understand the influence skin microbiota has on host–pathogen interactions, we describe the relationship of the amphibian skin microbiota of wild populations and the climatic factors across two years. In this study, we hypothesized that climatic conditions would influence the extent of Bd infection as well as the structure of skin bacterial communities. To examine this hypothesis we documented Bd infection status and described skin microbial structure of wild populations of a terrestrial cryptic frog (*P. loveridgei*) during two years across their breeding season that showed clear differences in precipitation levels. We also determined whether infection status correlated with changes in the diversity and structure of skin microbial communities.

## Materials and Methods

### Ethics Statement

This study was carried out in accordance with the recommendations of a Scientific Purpose permit issued by the Department of Environment and Resource Management (WITK10308811) Queensland, Australia. Griffith University Animal Ethics Committee (ENV/21/12AEC) approved the protocol.

### Field Sites

**FIGURE 1:**
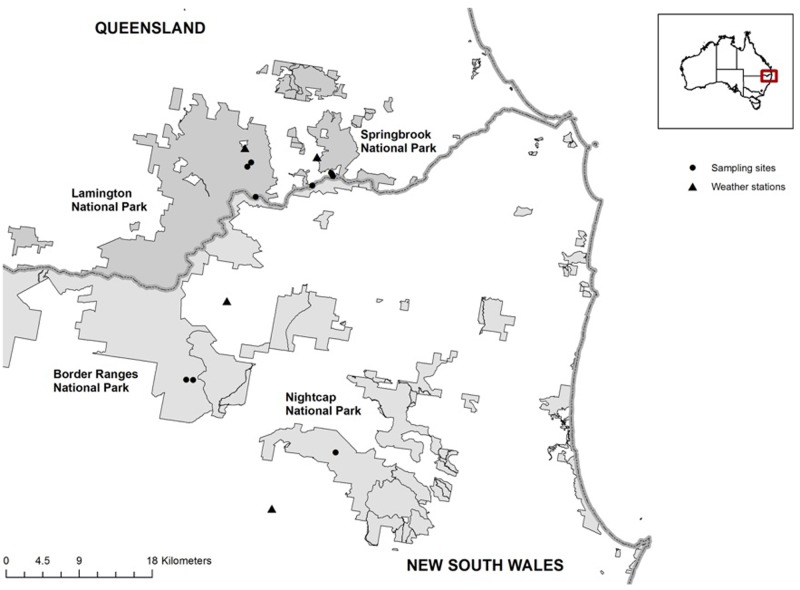
Study area in south–east Queensland and north–east New South Wales National Parks, indicating *P. loveridgei* survey sites (dots) throughout the range of the species and the nearest Australian Bureau of Meteorology stations (triangles).

Field surveys were done in four national parks that make up part of the Tweed Caldera Rim within Gondwana Rainforests of Australia World Heritage Area, in mid-eastern Australia (**Figure 1**). This region is characterized by subtropical rainforest vegetation and is dominated by a moist subtropical climate. At altitudes above 800 m above sea level (asl) a cool high-altitude subtropical rainforest occurs as rainfall is significantly augmented by fog-drip. In these mountain ranges the monthly summer average temperatures range between a maximum of 24.1°C and a minimum of 19.7°C, while winter temperatures range from 17.8 to 12.3°C, correspondingly. Rainfall in this area is seasonal with 65–70% of the annual total (average annual rainfall >3000 mm) occurring in the summer months (McDonald, 2010).

### Sample Collection

Surveys were conducted in two separate years during the frog species breeding season of each year (Australian spring and summer months) from November 2011 to January 2012 (here after year 1), and from November 2013 to January 2014 (here after year 2). Sample collection took place at nine sites covering the altitudinal and distribution ranges of *P. loveridgei*, including six sites in southeast Queensland (Springbrook and Lamington National Parks), and three sites in northeast New South Wales (Nightcap and Border Ranges National Parks). These sites were selected as they are highly occupied by the target species (MFL personal observation). The altitudes of the sites where frogs were encountered ranged from 680 to 1080 m asl (**Table 1**).

**Table 1:** Field surveys sites and dates of *P. loveridgei* across the species distribution range, across two years during the breeding seasons.

Site	National Park	Altitude (m)	Date sampled	Number of frogs
Bar Mt. picnic area	Border ranges	1083.1	November 2011; December 2013	13
Bar Mt.	Border ranges	977.1	December 2013; January 2014	13
Binna Burra 1	Lamington	892.8	November 2011	4
Binna Burra 2	Lamington	1020.6	November 2011; January 2012	4
Binna Burra 3	Lamington	859.9	November 2011	2
Mt. Nardi	Nightcap	683.4	December 2011; November and December 2013; January 2014	8
Best of all lookout	Springbrook	986.3	November and December 2011; November and December 2013	16
Bilborough 2	Springbrook	848.5	November 2011; December 2013; January 2014	7
Bilborough bridge	Springbrook	815.2	December 2013	5

Sample collection consisted of microhabitat surveys and intensive burrow searches performed by carefully excavating under leaf litter or rocks at sites where calling males were heard, with a minimum target of 10 frogs per site. *P. loveridgei* frogs, particularly males, were found sitting inside burrows. Call mimicry was sometimes used to incite males to call, thereby aiding the discovery of their location. Frogs were captured with freezer plastic bags and kept individually, and two skin swabs samples were collected following standard published protocols for Bd first and skin bacteria second (Brem et al., 2007; Hyatt et al., 2007; Kriger and Hero, 2007; Vredenburg et al., 2010). Before swabbing, frogs were rinsed with sterile distill water to eliminate transient bacteria (Rebollar et al., 2014). Swabbing consisted of moving a swab across the frog’s skin in a standardized way, using sterile rayon-tipped swabs (MW113; Lakewood Biochemical CO., United States), five strokes on each side of the abdominal midline, five strokes on the inner thighs of each hind leg, and five strokes on the foot webbing of each hind leg for a total of 30 strokes per frog (Kriger et al., 2006b; Vredenburg et al., 2010; Rebollar et al., 2014). Swabs were immediately placed in individually labeled microtubes and stored in a dry, cool place until transported back to the laboratory and stored at 4°C. To ensure consistency in swabbing technique all frogs were swabbed by MFL (Kriger et al., 2006b; Simpkins et al., 2014).

The morphological characteristics of all individuals, including body mass and size (snout-vent length, SVL), were recorded after skin samples were taken. All frogs were captured and handled at all times using individual, clean, unused freezer plastic bags. Additionally, all equipment used was washed with a solution of commercial bleach (1:9, bleach: water) and thoroughly cleaned between collection sites to prevent potential spread of the fungus pathogen (Phillott et al., 2010). Frog sampling was biased toward males as there sedentary calling behavior made them relatively more conspicuous than females. All burrows were individually marked to prevent resampling the same individual and to allow the release of individual frogs to their exact site of capture.

### Molecular Methods and Data Analyses for Bd Analyses

Real-time quantitative PCR (qPCR) analyses of the frog swabs were used to test for Bd infection, prevalence and intensity. Swabs were analyzed at San Francisco State University (San Francisco, California, United States), using the protocol described by Boyle et al. (2004) with the following changes: swab extracts were analyzed singly instead of in triplicate since use of singletons and triplicates has been found to give similar results in previous studies (Kriger et al., 2006a; Vredenburg et al., 2010). In addition, BSA was added to the qPCR master mix (1 μL BSA per reaction) (Garland et al., 2010).

Prevalence of Bd was calculated as the number of frogs that were Bd positive (when zoospore equivalents were ≥0.1) relative to the total number of frogs sampled. Infection intensity was defined as the number of zoospore equivalents per swab. Zoospore equivalents were estimated by multiplying the qPCR genomic value by 80, as DNA extracts from swabs were diluted 80-fold during extraction and qPCR (Briggs et al., 2010; Vredenburg et al., 2010).

Spearman correlations were used to assess the relationship between altitude, body size, weight, and the intensity of Bd infection. Mann–Whitney *U* test was used to assess the relationship between sex of the frog and the intensity of Bd infection. Analyses were conducted using R statistical software (R Core Team, 2015) specifically using the function cor.test (“spearman”) and wilcox.test().

### Molecular Methods and Sequencing for Bacterial Analyses

Whole genomic DNA was extracted from 72 swab frog samples using the DNeasy Blood and Tissue kit (Qiagen, Valencia, CA, United States) following the manufacturer’s instructions including a pretreatment with lysozyme included in the protocol titled: Pretreatment for Gram-Positive Bacteria (page 45). This pretreatment is recommended when trying to extract DNA from Gram-positive bacteria in addition to Gram-negative bacteria. DNA extracted from swabs was used to amplify the V4 region of the 16S rRNA gene using barcoded primers (F515/R806) and PCR conditions adapted from Caporaso et al. (2011). Amplicons were quantified using Quantifluor^TM^ (Promega, Madison, WI, United States). Composite samples for sequencing were created by combining equimolar ratios of amplicons from the individual samples, followed by cleaning with the QIAquick PCR clean up kit (Qiagen, Valencia, CA, United States). Barcoded composite PCR products were sent to the Dana Farber Cancer Institute’s Molecular Biology Core Facilities (Boston, MA, United States) for MiSeq Illumina sequencing using a 250 bp single read strategy.

### 16S Amplicon Data Processing

The 250 bp single reads were filtered and processed with the Quantitative Insights Into Microbial Ecology (QIIME) pipeline (Caporaso et al., 2010b). Sequences were de-multiplexed and filtered to retain high quality reads using the following filtering parameters: no *N* characters were allowed in retained sequences, no errors in barcode sequence were allowed, a minimum of five high quality consecutive base pairs were needed to include a read, and a maximum of five consecutive low quality base pairs were allowed before truncating a read. After filtering, 5,991,855 sequences were retained for the 72 samples.

The de-multiplexed data set was deposited in the NCBI sequence read archive (SRA) with the accession number SRR5957156 which is linked to biosample SAMN07501977 and bioproject PRJNA398139.

De-multiplexed and filtered sequences were clustered into operational taxonomic units (OTUs) at a sequence similarity threshold of 97% with the UCLUST method (Edgar, 2010). Sequences were matched against the Greengenes database released in May 2013 (McDonald et al., 2012), and those that did not match were clustered as *de novo* OTUs at 97% sequence similarity. Taxonomy was assigned using the RDP classifier (Wang et al., 2007) and the Greengenes database. Representative sequences were aligned to the Greengenes database with PyNAST (Caporaso et al., 2010a), and a ML phylogenetic tree was constructed with FastTree 2 (Price et al., 2010). The OTU table was filtered using a minimum cluster size of 0.001% of the total reads as suggested by Bokulich et al. (2013). Samples were rarefied according to the sample with lower number of reads (i.e., 25,605 reads). The final rarefied OTU table had 7,673 OTUs including a total of 1,843,560 reads.

### Bacterial Skin Data Analysis and Statistics

To describe the diversity of host skin bacteria in *P. loveridgei* across two years in the breeding season Shannon diversity index was calculated for all 72 samples. ANOVA and *post hoc* Tukey’s tests were carried out to determine differences between samples taken every month during the two years inbreeding seasons. Stacked bar charts of bacterial taxa at the class level were obtained to show the mean relative abundance of the 10 most abundant taxa on each month during the two years in the breeding seasons.

Weighted Unifrac and distance matrices were used to calculate the beta diversity and were visualized with a principal coordinates analysis. Differences in beta diversity between the two years samples and between Bd positive and negative samples were tested with non-parametric analysis of variance based on 999 permutations (PERMANOVA) using the software PRIMER-E (Clarke and Gorley, 2006). Alpha and beta diversity metrics and relative abundance comparisons at the class level were obtained using QIIME (Caporaso et al., 2010b). Analysis of multivariate homogeneity of group dispersions was calculated to determine whether skin communities from different breeding seasons had different dispersion values using the function betadisper in vegan package in R (Oksanen et al., 2015).

To determine the bacterial taxa that most likely explain differences between the two years, we used the linear discriminant analysis (LDA) effect size (LEfSe) method (Segata et al., 2011). Classes were defined as years 1 and 2, and subclasses corresponded to the months per breeding season. OTUs with LDA scores >2.0 were considered informative based on previous studies (Segata et al., 2011; Clemente et al., 2015; Rebollar et al., 2016a). Lefse was calculated using Galaxy (Afgan et al., 2016) implemented at the Huttenhower Lab^1^.

To determine if Bd infection intensities were correlated with bacterial relative abundance across frog samples we calculated Spearman correlations with false discovery rate (Benjamini–Hochberg) to account for multiple comparisons using R (R Core Team, 2015) using the functions cor.test(“spearman”) and p.adjust(“BH”). To perform this analysis we only included the data from year 2 since on year 1 Bd was not detected and the bacterial community was different than on year 2. Correlation values with *p* < 0.05 were considered significant. Wilcoxon Rank Sum Test in R (R Core Team, 2015), using the function wilcox.test(), was calculated to compare the relative abundance of bacterial taxa that had significant Spearman correlations between Bd-positive and Bd-negative samples.

## Results

### Bd Infection Status of *Philoria loveridgei*

We detected Bd infection in seven individuals of 72 *P. loveridgei* tested (8 females and 64 males), with an overall prevalence of 9.7% (*n* = 7, 6 males and 1 female), across the two years during the breeding season (year 1: 2011/2012 and year 2: 2013/2014). The seven frogs infected were all sampled in year 2 (*n* = 55) while year 1 yielded none (*n* = 17). The pathogen was found only at two of the nine sites surveyed, both located in the southern limits of *P. loveridgei’s* distribution range in New South Wales (**Figure 1**). The majority of infected frogs (*n* = 6) were from one site located in Border Ranges National Park (Bar Mt area), with a site infection prevalence of 35.3%. The only other Bd infected frog was from Nightcap National Park, with a site prevalence of 12.5% (**Table 2**). These sites have a difference in altitude of 300 m asl; the Nightcap site was the lowest area surveyed in this study. Altitude, however, was not significantly correlated with Bd infection (ρ = -0.126, *P* = 0.290, *n* = 72).

**Table 2:** Disease prevalence and infection loads (Bd zoospore equivalents) of individual *P. loveridgei* per site and per year sampled (year 1 = 2011/2012 and year 2 = 2013/2014).

Site	Number of frogs (infected)	Year 1 Bd prevalence	Number of frogs (infected)	Year 2 Bd prevalence	Infection loads
Bar Mt. picnic area	1 (0)	0	12 (0)	0	0
Bar Mt.	0 (0)	0	**13 (6)**	**35.3%**	**19.1 ± 28.6**
Binna Burra 1	4 (0)	0	0 (0)	0	0
Binna Burra 2	4 (0)	0	0 (0)	0	0
Binna Burra 3	2 (0)	0	0 (0)	0	0
Mt. Nardi	1 (0)	0	**7 (1)**	**14.3%**	**124.8**
Best of all lookout	4 (0)	0	12 (0)	0	0
Bilborough 2	1 (0)	0	6 (0)	0	0
Bilborough bridge	0 (0)	0	5 (0)	0	0

*Sites where Bd infection was detected are highlighted in bold.*

We found that overall, infection intensity was relatively low with an average of 34.2 ± 47.7 zoospore equivalents (mean ± standard deviation), ranging from as low as 0.79 to 124.8 zoospore equivalents per individual. The frog presenting the highest infection intensity (124.8 zoospore equivalents) was from the Nightcap site (**Table 2**).

Male frogs weighed an average 2.2 ± 0.7 g and had a mean SVL of 25.6 ± 0.3 mm (mean ± standard deviation). As expected, female frogs were bigger than male frogs weighing an average 2.8 ± 0.6 g and had a mean SVL of 28.8 ± 0.2 mm. No association was found between sex of the frog and infection status (Mann–Whitney *U* = 250, *P* = 0.848, *n* = 72). Additionally, size and weight of frogs were not significantly correlated with Bd intensity (size; ρ = 0.130, *P* = 0.278, *n* = 72; weight; ρ = -0.082, *P* = 0.496, *n* = 72). No clinical signs of chytridiomycosis were observed in any of the frogs surveyed (Berger et al., 1998, 2005).

### Temporal Variation of the Skin Bacterial Community Structure of ylum Proteobacteria was the most a

A total of 7,673 OTUs were identified from 72 frog samples collected during three months across two years in the breeding season. Overall, phylum Proteobacteria was the most abundant (49.9%) across all samples, followed by Actinobacteria (13.37%), Acidobacteria (7.71%), Verrucomicrobia (4.74%), and Planctomycetes (3.16%).

**FIGURE 2:**
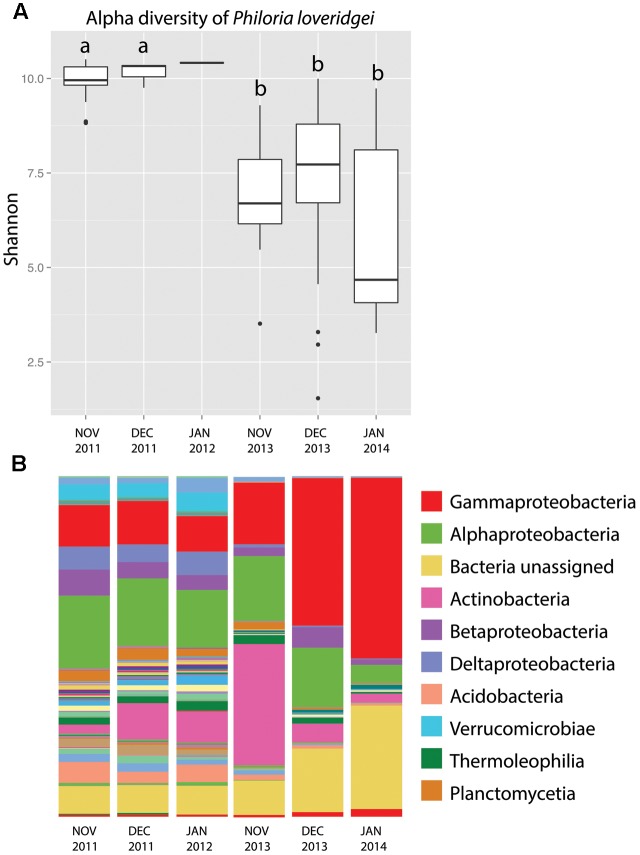
Skin bacterial community structure of *P. loveridgei* across time in two breeding seasons. **(A)** Alpha diversity (Shannon) of *P. loveridgei* [ANOVA *F*(5,66) = 8.215, *P* = 4.46 × 10^–6^]. Different letters (a and b) signify statistically significant differences across months, as indicated by a Tukey’s *post hoc* test. **(B)** Stacked bar chart of mean relative abundances of bacterial taxa (class level) of *P. loveridgei*. Colored bars (legend) indicate the 10 most abundant taxa.

According to Shannon index estimates, skin bacterial alpha diversity was significantly higher in *P. loveridgei* skin samples from year 1 in comparison to year 2 [**Figure 2A**; ANOVA *F*(5,66) = 8.215, *p* = 4.46 × 10^-6^]. These diversity values indicate significant differences in bacterial community richness and relative abundance between months of different years but not between months within the same year.

**FIGURE 3:**
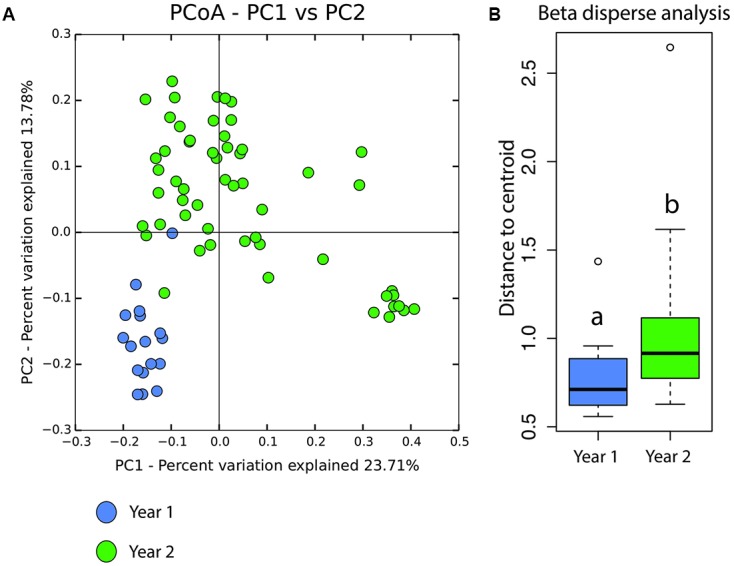
**(A)** Beta diversity of *P. loveridgei* between year 1 (blue) and year 2 (green). Principal coordinate analysis is based on weighted Unifrac distances [ADONIS *F*(1,71) = 5.8841, *P* = 0.001]. **(B)** Analysis of multivariate homogeneity of group dispersions (variance) of *P. loveridgei* skin microbial communities. Box plot of distances to each centroid’s group. Different letters (a and b) signify statistically significant differences between year 1 and year 2 [ANOVA *F*(1,70) = 5.9827, *P* = 0.01696].

Specifically, at the class level, Alphaproteobacteria were more abundant in year 1 while Gammaproteobacteria were more abundant in year 2 (**Figure 2B**). Beta diversity showed significant differences between these two years as seen with Bray–Curtis distances [Adonis test: *F*(1,71) = 5.8841, *p* = 0.001]. In addition, beta disperse analysis indicated a greater variance among samples from the 2nd year [**Figures 3A,B**; ANOVA *F*(1,70) = 5.982, *p* = 0.016].

With the aim to determine which OTUs significantly differed between years, a LEfse analysis was performed. This analysis determined that 569 out of 7673 OTUs (7.4% of the total OTUs) were significantly different and more abundant in year 1. In contrast, in year 2, only seven OTUs from the classes Alphaproteobacteria, Gammaproteobacteria, and Bacilli had a higher relative abundance (Supplementary Table 1).

### Climatic Differences between Years

**FIGURE 4:**
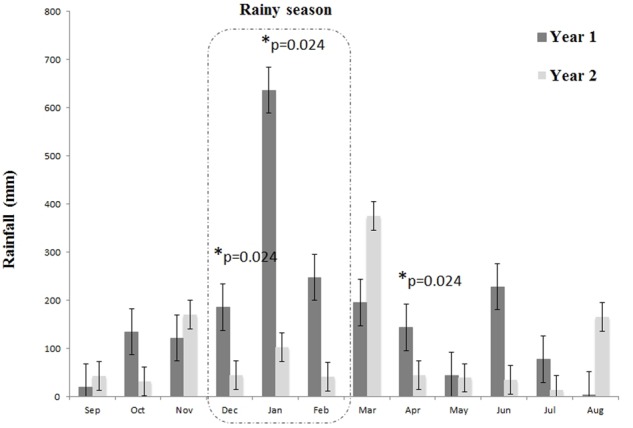
Rainfall levels during the two study years including the breeding seasons were *P. loveridgei* frogs were surveyed. Asterisks indicate months were year 1 was significantly higher than year 2 according to *t*-test.

Differences between years 1 and 2 may be caused by several factors, but we found that rainfall patterns varied significantly between the two breeding seasons evaluated in this study (**Figure 4**). Specifically December and January, the rainy month of the year, showed significantly higher mean monthly rainfall in year 1 when compared to year 2 (Dec: *ρ* = 0.024; Jan: *ρ* = 0.024) (**Figure 4**). The 1st year being a “La Niña” year, which was characterized by heavy summer rainfalls, compared to a neutral phase year for the 2nd year. Furthermore, the “La Niña” year of the 1st year of the study (2011) followed a stronger “La Niña” event of 2010, bringing the highest two years of Australian rainfall on record, including heavy rainfall at the study area. Additionally, 2010 and 2011 were the coolest years recorded since 2001 due to the two consecutive “La Niña” events (CSIRO, 2012). Year 2 was a neutral year and Australia’s warmest year on record, with widespread warmth throughout the year, and below average rainfall. Particularly, summer rainfall at the study area was in the lowest 10% of records, with some small areas recording their lowest summer rainfall on record. Additionally, year 2 (2013) was preceded by 2012 climatic patterns which reflected a shift from a “La Niña” year to a neutral year, but warmer than average (CSIRO, 2014).

### Correlation between Bd Infection Intensity and Skin Bacterial Diversity in Two Breeding Seasons

**FIGURE 5:**
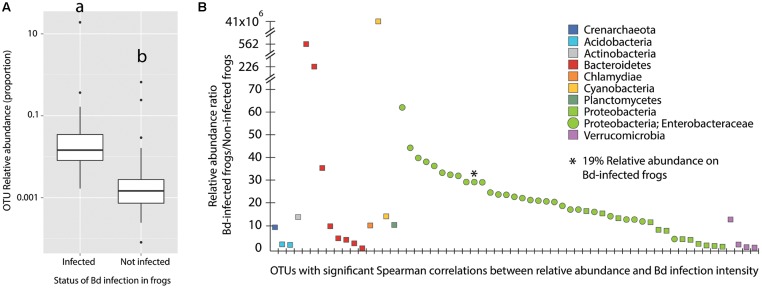
**(A)** Relative abundance of 67 bacterial OTUs with a significant Spearman correlation (Bd loads vs. OTUs relative abundance) on Bd-infected individuals in comparison to non-infected individuals. Different letters (a and b) signify statistically significant differences, as indicated by a Wilcoxon rank sum test (*W* = 4102, *p*-value < 2.2e^–16^). **(B)** Relative abundance ratio (Bd-infected frogs/Non-infected frogs) of OTUs with a significant Spearman correlation. *Y*-axis was adapted to fit the highest values.

To determine the correlation between Bd infection status and the skin bacterial community structure we only analyzed the data from year 2, as Bd was not detected in year 1 and there were significant differences on the bacterial communities between years 1 and 2. We found no significant differences between Bd status (infected/not infected) based on bacterial alpha [Supplementary Figure 1A; ANOVA *F*(1,70) = 2.579, *p* = 0.113] and beta diversity estimates [Supplementary Figure 1B; Adonis test: *F*(1,71) = 1.707, *p* = 0.07]. However, when analyzing Bd infection loads we found positive significant Spearman correlations between Bd infection intensity and the relative abundance of 67 bacterial OTUs (Supplementary Table 2). These OTUs had a higher relative abundance in infected individuals than in non-infected individuals (**Figure 5A**) and Wilcoxon rank sum test indicated significant differences between these two groups (*W* = 4102, *p* < 2.2 × 10^-16^). OTUs with a higher relative abundance in Bd-infected individuals were classified in eight different bacterial phyla and one archaeal phylum (**Figure 5B**). Moreover, of the 67 OTUs, 41 (63%) were from the phylum Proteobacteria and 29 of these OTUs (45%) were from the family Enterobacteriaceae. Interestingly, one specific OTU from the family Enterobacteriaceae accounted for 19% of the relative abundance in Bd-infected frogs (**Figure 5B**).

## Discussion

In this study, we found differences on Bd status and skin microbial communities on *P. loveridgei* populations across the two years in the breeding season. Overall, even though sampling sites differed on altitude, temporal variation across the two breeding seasons was the factor that better explained the changes in Bd infection status and bacterial community structure of wild populations of *P. loveridgei*. Importantly, these two breeding seasons differed dramatically on their climatic conditions, with the 1st year being part of “la Niña year” characterized by high precipitation levels.

We found Bd in two wild populations of *P. loveridgei* at low (683 m) and mid-high (977 m) altitude subtropical rainforest sites in mid-eastern Australia in only one year of the two that were surveyed. Our results indicate low infection intensity and low disease prevalence in this frog species, however, we still lack knowledge on the impact that this disease may have on *P. loveridgei* wild populations. Our findings of low infection intensity are in agreement with those observed in two other Australian terrestrial species *C. ornatus* and *A. darlingtoni*, where Bd infection was also relatively low (31 and 189 zoospore equivalents, respectively) (Kriger and Hero, 2006, 2007). *B. dendrobatidis* presence and infection intensity on *P. loveridgei* had not been reported before, yet our study suggests that *P. loveridgei* populations are likely to be exposed to the pathogen throughout their altitudinal range. Moreover, some of the subtropical rainforest areas and lowlands that bordered our sites were previously known to have undergone disease outbreaks, with various stream breeding frog species found to be infected with Bd (Berger et al., 2004; Kriger and Hero, 2008; Narayan et al., 2014). Furthermore, disease dynamics in *P. loveridgei* may depend on additional variables associated to changes in climatic conditions, such as temperature and precipitation as reported for other species (Longo et al., 2010). For example, prevalence was related to annual environmental changes in *Litoria wilcoxii* (a stream breeding frog), with higher prevalence levels in winter and early spring months (Kriger and Hero, 2008). Similarly, a multi-species study of wild frogs from Queensland and New South Wales reported a higher incidence of chytridiomycosis during winter months compared to other seasons of the year, when mean temperatures are low and in the optimal growth range of Bd compared with summer temperatures which are higher and can restrict Bd (Berger et al., 2004). Interestingly, a study on wild populations of two direct-developing frogs, *E. coqui* and *E. portoricensis*, found an association between Bd prevalence and infection intensity and seasons (Longo et al., 2010). The authors proposed a lower infection intensity in the wet and warm season and a higher in the dry and cool, as a result of the climatic differences but also of differential behavioral patterns (Longo et al., 2010).

The Bd infection intensity and prevalence patterns we observed in *P. loveridgei* suggest an enzootic state between the host and the pathogen. Despite finding populations of *P. loveridgei* infected with Bd, we found no clinical signs of chytridiomycosis or death related to this illness, nor have they been reported elsewhere. Additionally, no population declines related to Bd have been documented for any Australian direct-developing amphibian species (Hines et al., 1999; Hero et al., 2006; Hauselberger and Alford, 2012) in contrast with reports from other parts of the world (Bell et al., 2004; Burrowes et al., 2004; Lips et al., 2006; Longo and Burrowes, 2010; Longo et al., 2013). These results imply that Australian direct-developing amphibian species are surviving in an enzootic state with Bd perhaps through an innate immune response that could be providing them with protection against Bd, or by harboring beneficial skin microbiota. Brem and Lips (2008) suggest that terrestrial amphibians may share similar skin microbial fauna that provides them with protection to Bd and in turn make them less susceptible as a group. A study of four Australian frogs showed higher Bd resistance in species with powerful skin peptide defenses when experimentally infected with Bd (Woodhams et al., 2007b). Other studies have shown the inhibitory role of skin bacteria in the growth of Bd (Harris et al., 2006, 2009; Woodhams et al., 2007a; Walke et al., 2011). In wild *P. loveridgei* populations, higher Bd infection intensity may be found in seasons of the year we could not sample (e.g., cool and dry winter). Individuals of this fossorial cryptic frog species remain in their underground burrows during non-breeding months, making sampling extremely difficult. Because high Bd infection intensity implies susceptibility to chytridiomycosis (Vredenburg et al., 2010), the low level we discovered in this frog species could suggest resistance or tolerance to the infection, due to a more effective immune system, antimicrobial skin peptides or the presence of skin microbiota (Harris et al., 2009; Rollins-Smith, 2009).

We found differences in skin bacterial community structure across the two years in the breeding seasons. A high diversity of bacteria was found during the rainy and cool year (year 1: 2011), which was characterized by a higher abundance of Alphaproteobacteria. This was followed by an overall decrease on diversity during year 2 (2013) and the increase in abundance of Gammaproteobacteria and Actinobacteria. Interestingly, the 2nd year was the only time where Bd was detected on these frogs. The decrease of bacterial diversity has been correlated with the presence of Bd in previous field studies (Jani and Briggs, 2014; Rebollar et al., 2016b; Longo and Zamudio, 2017b). In *Rana sierrae* wild populations, Bd infection was strongly correlated with bacterial community composition with several taxa decreasing in abundance (Betaproteobacteria, Gammaproteobacteria, Actinobacteria and also a few members from the Acidobacteria and Alphaproteobacteria) (Jani and Briggs, 2014). Similarly, on a field survey of the terrestrial Panamanian frog *Craugastor fitzingeri*, Bd presence seemed to influence skin bacterial community structure with the Bd-naïve site dominated by OTUs from the genus *Acinetobacter* (Gammaproteobacteria) compared to the Bd-endemic sites dominated by *Pseudomonas* (Gammaproteobacteria), *Cellulomonas* and *Sanguibacter* (both Actinobacteria) (Rebollar et al., 2016b). Proteobacteria were also the most abundant phyla in skin samples of *R. sphenocephala* (Holden et al., 2015). Interestingly, this study found isolates of Proteobacteria and Actinobacteria that are able to inhibit the growth of Bd.

The changes in microbial diversity observed between breeding seasons may be due to several factors including climatic fluctuations (precipitation and temperature) as seen in previous studies (Longo and Zamudio, 2017a,b; Muletz Wolz et al., 2017; Sabino-Pinto et al., 2017). Climatic conditions may in turn influence host behavior, host susceptibility to pathogens, as well as Bd infection capacity (Longo et al., 2010; Longo and Zamudio, 2017a,b). For example, aggregation behavior was observed in two direct-developing frogs, *E. coqui* and *E. portoricensis*, during the dry season, while dispersion of the host occurred during the wet season (Longo et al., 2010). Heavily infected terrestrial frogs may remain inactive and hidden in fossorial retreats under dry and cool conditions, hindering their detection. In contrast, during warm and wet seasons frogs have lower infection levels and are more active in the forest, increasing the probability of sampling an infected frog and yielding higher prevalence estimates (Longo et al., 2010). Furthermore, climatic conditions can also lead to changes in microbial communities and in turn Bd infection and prevalence (Longo and Zamudio, 2017a). In another study of the two direct-developing frogs, Bd infection may have been limited during the warm and wet season by recruitment of putatively beneficial bacteria followed by a return to pre-infected levels of bacteria richness and diversity was observed (Longo and Zamudio, 2017a). Additionally, during the cool and dry season Bd infection increased through time and bacterial diversity did not change (Longo and Zamudio, 2017a). Overall, host-pathogen interactions may be strongly influenced by climatic fluctuations as seen in previous studies. Thus, we hypothesize that changes in climatic conditions between the two years in the breeding seasons that we surveyed could explain the presence of Bd and the decrease of skin microbial diversity. Thus, climatic changes may directly influence the skin bacteria community as well as the success of Bd to infect host, but also there might be an interaction occurring between the skin symbiotic community structure and the presence of the pathogen on the skin. However, it is important to consider that, besides climate, our results could be explained by other factors that we did not consider in this study.

Our study identified a group of 67 bacterial taxa that were enriched on the frogs from year 2, which were characterized by harboring higher Bd loads (significant positive Spearman correlations). A great proportion of these OTUs belonged specifically to the family Enterobacteriaceae. Interestingly, a data base for culturable Bd inhibitory bacteria published by Woodhams et al. (2015) showed that 94% of the culturable bacteria from this family inhibit Bd growth. Our field survey suggests that the increase in the abundance of Enterobacteriaceae OTUs might be related to Bd infection, e.g., these bacteria may be recruited to inhibit the pathogen or may be a consequence of Bd infection on the skin. To further test this hypothesis additional experimental studies will need to be performed.

Overall, our study contributes on the understanding of the role of temporal variation on Bd infection and skin microbial community structure. However, in order to fully determine an effect of the climatic conditions on the skin microbial structure and pathogen infection, a long term study is essential. This study supports previous findings showing that Bd estimates and microbial community description are not static and therefore require multiple temporal and spatial samplings to fully understand the ecological interactions occurring between the host, the pathogen and bacterial symbiotic communities.

## Author Contributions

MFL contributed to the conception of the idea and design of the research, and carried out the fieldwork. MFL, ER, and VV contributed with laboratory analysis. MFL and ER analyzed the data with advise from RH and J-MH. All authors contributed with the interpretation of the data. MFL and ER wrote the manuscript and all authors provided critical feedback.

## Conflict of Interest Statement

The authors declare that the research was conducted in the absence of any commercial or financial relationships that could be construed as a potential conflict of interest.

## Footnotes

1 https://huttenhower.sph.harvard.edu/galaxy/
