# Supplementary material for: Temporal Variation of the Skin Bacterial Community and Batrachochytrium dendrobatidis Infection in the Terrestrial Cryptic Frog Philoria loveridgei
Source: Front Microbiol. 2017 Dec 22;8:2535. doi: 10.3389/fmicb.2017.02535 (PMC5744006; doi:10.3389/fmicb.2017.02535)
Supplement: Supplementary file 3 [file Data_Sheet_1.PDF]

Supplementary Material

Temporal variation of the skin bacterial community and *Batrachochytrium dendrobatidis* infection in the terrestrial cryptic frog *Philoria loveridgei*

Mariel Familiar López*, Eria A. Rebollar, Reid N. Harris, Vance T. Vredenburg and Jean-Marc Hero

*** Correspondence:** Mariel Familiar López: mariel.familiarlopez@griffithuni.edu.au

1 Supplementary Figures and Tables

1.1 Supplementary Figures

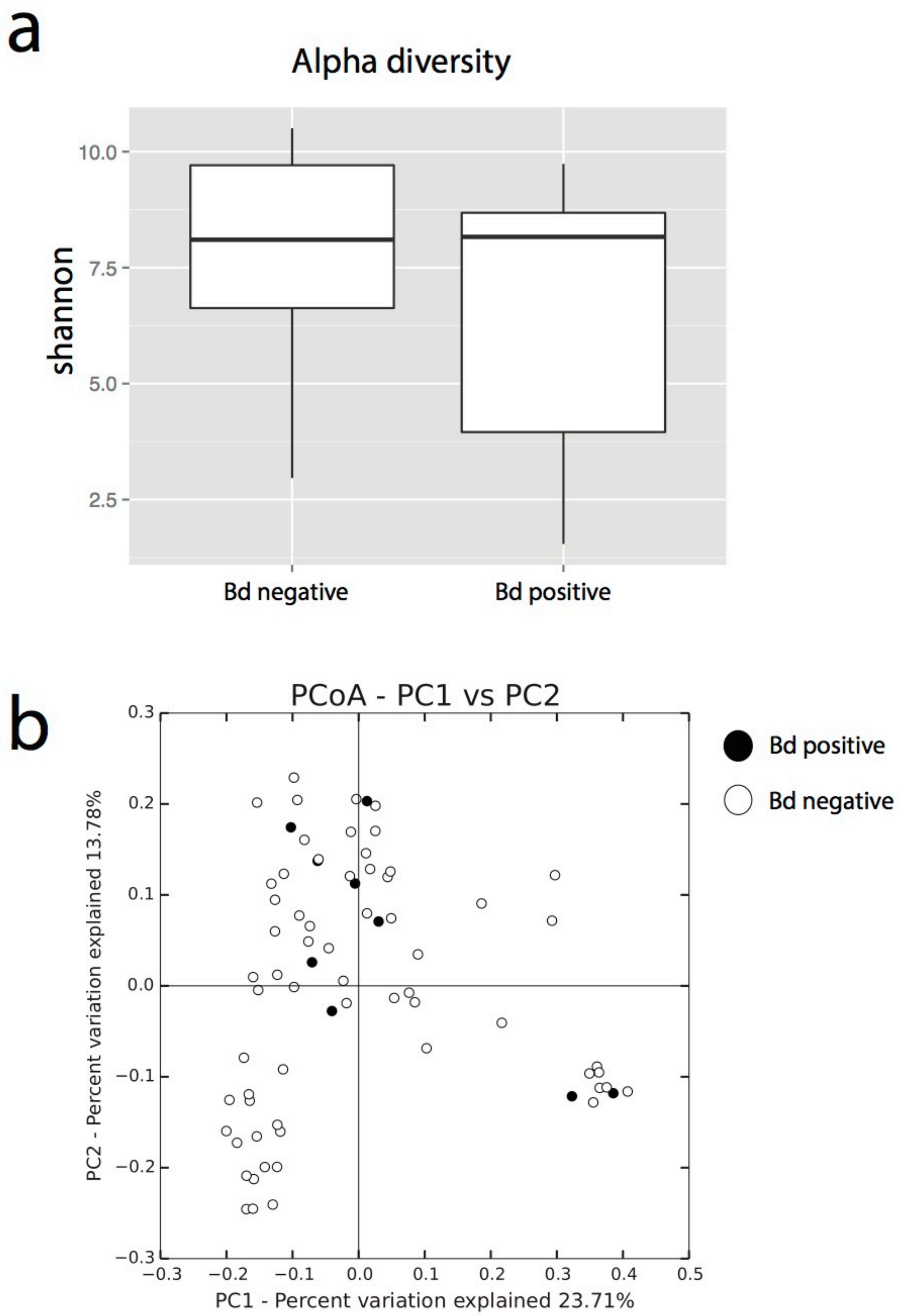

Supplementary Figure 1 a) Beta diversity of bacterial communities according to Bd status (Bd infected and not infected). Black circles (positive) and white circles (negative) (PERMANOVA Pseudo-F(1,71) =1.7073, P=0.07) **b)** Alpha diversity (Shannon) of *P. loveridgei* (ANOVA F(1,70) = 2.579, P=0.113) of infected and not infected individuals.

1.2 Supplementary Tables

Supplementary Table 1 List of OTUs that were significantly different between breeding seasons according to Lefse analysis.

Supplementary Table 2 List of 67 OTUs significantly correlated with Bd infection intensity based on Spearman correlation.
